# Application value of TPAS staining technique for cervical exfoliated cells in cervical cancer screening

**DOI:** 10.1186/s13000-025-01744-w

**Published:** 2025-12-22

**Authors:** Guo Chen, Wenli Zhang, Yuchen HuYan, Yipching Yang, Wensheng Li, Guoqiang Feng, Zifan Lu

**Affiliations:** 1 Translational Medical Center, Shaanxi Provincial People’ s Hospital, Xi’an, China; 2 University of California, Berkeley, USA; 3 Department of Pathology, Shaanxi Provincial People’ s Hospital, Xi’an, China; 4 Key Laboratory of Pesticide and Chemical Biology of Ministry of Education, College of Chemistry, Central China Normal University, Wuhan, China

**Keywords:** Cervical cancer screening, TPAS staining, Liquid-based thin-layer cell testing(TCT), Human papillomavirus testing(HPV)

## Abstract

**Background:**

Recent studies have shown that tumor cell membranes exhibit lower polarity than normal cell membranes, a characteristic that can be harnessed for cancer diagnosis. TPAS (viscosity-responsive plasma membrane probe), a recently developed staining method using cell membrane polarity probes, may selectively visualize cervical cancer cells by targeting membrane polarity differences, offering a potential new approach for cervical cancer screening.To investigate the diagnostic value of TPAS staining combined with liquid-based thin-layer cytological testing (TCT) and human papillomavirus (HPV) testing in detecting cervical cancer.

**Methods:**

A total of 100 patients with suspected cervical precancerous lesions from the People’s Hospital of Shaanxi Province between May 2024 and May 2025 were studied. All patients underwent TPAS testing, HPV testing and TCT. Biopsy results were the gold standard for evaluating positivity rates and diagnostic values of the tests, both individually and in combination.

**Results:**

Among the 100 participants, the positive rates of the tests were as follows: TPAS detection rate was 86.7%, HPV detection rate was 80%, TCT rate 66.7%, and TCT + HPV rate was 35.00%. The combined TPAS + HPV testing showed higher accuracy (72.00%) and sensitivity(70.6%) than TCT + HPV (58.0%)and (54.1%), and the differences were statistically significant ((χ2 = 14.00,*P* = 0.0002).

**Conclusion:**

TPAS combined with HPV testing has high specificity, sensitivity and accuracy, making it a promising approach for cervical cancer diagnosis.

## Introduction

Cervical cancer is one of the most prevalent malignant tumors in women. Its early symptoms are often indistinct, and patients typically seek medical attention when the cancer has progressed to an advanced stage, precluding surgical intervention. This explains why cervical cancer exhibits high incidence and mortality rates [1]. Persistent infection with high-risk human papillomavirus (HPV) serves as a key driver in the development of cervical precancerous lesions and cervical cancer [2]. Effective early screening is crucial for reducing the disease burden [3]. In China, the primary cervical cancer screening methods are liquid-based cytology testing (TCT), HPV testing, and their combination. However, these approaches suffer from high costs, complex procedures, and reliance on professional pathologists or laboratory technicians. Liquid-based cytology (LBC) represented by the ThinPrep test and HPV DNA genotyping have emerged as the most validated noninvasive strategies for cervical cancer screening [4, 5]. Cumulative evidence has established persistent HPV infection as a pivotal etiological factor, prompting guidelines to endorse HPV DNA testing as the primary screening modality for women over 30 or those with indeterminate ThinPrep results [6]. Notably, operator-dependent sample collection and morphological interpretation in TCT contribute to false-positive/negative outcomes, especially for atypical squamous cells of undetermined significance (ASCUS) [7, 8]. Additionally, transient HPV carriers may undergo unnecessary interventions due to false-positive DNA test results [9]. While co-testing with LBC and HPV DNA enhances sensitivity, it fails to improve diagnostic specificity [10]. These limitations highlight an urgent need for innovative co-screening strategies to facilitate early detection of cervical intraepithelial neoplasia (CIN).

Against this backdrop, there is an imperative to develop a rapid, convenient screening method for widespread application. Given China’s national context, identifying a new cervical cancer screening approach that is stable, safe, and easy to perform is essential [8].

In our investigation, we innovatively applied a novel viscosity-sensitive cell membrane probe, TPAS, for detecting exfoliated cervical epithelial cells. TPAS is a fluorescent dye with aggregation-induced emission (AIE) characteristics. Its unique chemical structure—incorporating triphenylamine (TPA) as an electron-donating unit, combined with the strong electron-withdrawing effect of acetonitrile pyridinium salt and the hydrophilic sulfonate group—forms an amphiphilic D-π-A conjugated structure. This design ensures good water solubility and effective cell membrane targeting, enabling accurate detection of microenvironmental changes in cell membranes [11].

Experimentally, TPAS exhibited distinct fluorescence responses in different viscosity environments. In low-viscosity solutions, TPAS’s rotor moiety rotates freely, causing energy loss via non-radiative transitions and weak fluorescence. In high-viscosity environments, however, molecular rotation is restricted, leading to significantly enhanced fluorescence intensity. Notably, TPAS fluorescence intensity shows a linear correlation with viscosity, providing a reliable basis for evaluating cell membrane viscosity differences through fluorescence signals. Moreover, the AIE property of TPAS avoids fluorescence quenching during bioimaging, demonstrating excellent photostability for long-term dynamic membrane imaging.

When applied to exfoliated cervical epithelial cells, TPAS successfully differentiated between normal and cancerous cells. Results showed that TPAS selectively labeled the membranes of cancerous cervical epithelial cells, with fluorescence intensity significantly higher than that of normal cells—consistent with its performance in tumor-normal cell discrimination reported in literature. This finding suggests that cervical cancer cells exhibit higher membrane viscosity than normal cells, indicating that TPAS fluorescence changes can serve as a reliable marker for detecting such changes and offer a new approach for early cervical cancer screening.

This study comparatively analyzed the diagnostic value of three screening methods—TPAS detection, TCT and HPV testing—along with their combined use, in the primary screening of cervical lesions.

## Materials and methods

### Clinical date

A total of 100 women undergoing cervical lesion screening at Shaanxi Provincial People’s Hospital between May 2024 and May 2025 were enrolled. All participants voluntarily participated and signed informed consent forms. They had a history of sexual intercourse, no history of cervical cancer or precancerous lesions, and no history of cervical surgery.TPAS staining, TCT, and the HR-HPV test were used for screening for cervical cancer. The screening results were collected, and pathological examinations of cervix tissue were performed using the three-step criteria. The study protocol was approved by the Medical Ethics Committee (Approval No.: R043), which accorded with the ethical standards formulated in the Helsinki Declaration.

### TCT test

The cervical squamocolumnar junction (SCJ) served as the target site for specimen collection using a dedicated ThinPrep cytology brush. The brush was inserted into the cervical canal and rotated 3–5 times in a clockwise direction to ensure adequate cellular sampling. Immediately after collection, samples were transferred to a preservative solution within a sterile container and stored at ambient temperature (20–25 °C) until processing, to maintain cellular integrity.All cytological slides were independently reviewed by two board-certified gynecologic pathologists with ≥ 10 years of experience, following the 2001 Bethesda System for Reporting Cervical Cytology. Diagnostic criteria were standardized prior to the study, with discrepancies resolved through consensus review. Results were categorized into: Negative findings: Negative for intraepithelial lesion or malignancy (NILM); Positive findings: Atypical squamous cells of undetermined significance (ASCUS), atypical squamous cells cannot exclude high-grade lesion (ASC-H), low-grade squamous intraepithelial lesion (LSIL), high-grade squamous intraepithelial lesion (HSIL), and squamous cell carcinoma (SCC).

### HR-HPV test

The HR-HPV genotyping detection was performed by polymerase chain reaction (PCR) technology using the Kaipu Biotech 21-type genotyping detection kit (Guangdong Kaipu Biotechnology Co., Ltd., Chaozhou, Guangdong Province, China.). After exposing the cervix, the cervical surface secretions were wiped clean with a cotton swab. An HPV conical sampling brush was inserted 1–1.5 cm into the cervical canal, rotated clockwise 5–10 times, and then removed and placed in the preservation solution. The HPV genotyping detection was conducted by laboratory physicians. High-risk types including HPV16, 18, 31, 33, 35, 39, 45, 51, 52, 53, 56, 58, 59, 66, and 68 were defined as positive for HPV detection.

### Colposcopy

Colposcopy is indicated when any of the following tests yield a positive result: TPAS, TCT, or HPV testing. During the procedure, if cervical lesions are visualized, a cervical biopsy is performed. The cervix is first exposed, and cervical surface secretions are removed using a cotton ball moistened with normal saline. Subsequently, the cervix is soaked with 5% acetic acid for approximately 1 min to observe any changes in cervical tissue. Lugol’s iodine is then applied to the cervical surface, and the staining pattern is carefully examined. In cases where the colposcopic findings are unsatisfactory, endocervical curettage is carried out. The obtained tissue samples are submitted for pathological examination. The histopathological results of the cervical biopsy serve as the gold standard, with CIN2 and above being considered positive.

### Calculation methods

Sensitivity (true-positive rate): Pathological diagnosis was used as the gold standard, and the pathological and screening results were both positive. Sensitivity is calculated as follows: sensitivity = number of true positives/(number of true positives + number of false negatives)×100%. Specificity (true-negative rate) refers to the proportion of disease-free cases diagnosed as disease-free according to the screening results. Specificity is calculated as: specificity = number of true negatives/(number of true negatives + number of false positives)×100%. Positive predictive value (PPV) = number of true positives/(number of true positives + number of false positives)×100%. Negative predictive value (NPV) = number of true negatives/(number of true negatives + number of false negatives)×100%. Accuracy = [(number of true positives + number of true negatives)/total number of cases]×100%. False-positive rate = number of false positives/(number of true negatives + number of false positives)×100%. Youden index = (sensitivity + specificity) – 1.

## Results

### Detection rates by histopathological diagnosis and test method

Overview of the data included in this screening study: 100 patients with complete data from TCT, HR-HPV test, TPAS staining, and pathological diagnosis via cervical biopsy were included in this study. The age range of the 100 women was 43.1 ± 6.8 years (20–73 years). According to the pathological diagnostic results, there were 66 cases of normal cervix, 19 cases of LSIL (CIN1), 13 cases of HSIL (CIN2 and CIN3), and 2 cases of cervical cancer. There were 15 cases of HSIL (CIN2+, namely CIN2 and above), and 7 cases of HSIL (CIN3+, namely CIN3 and above). The screening results are shown in Table 1.

**Table 1.**
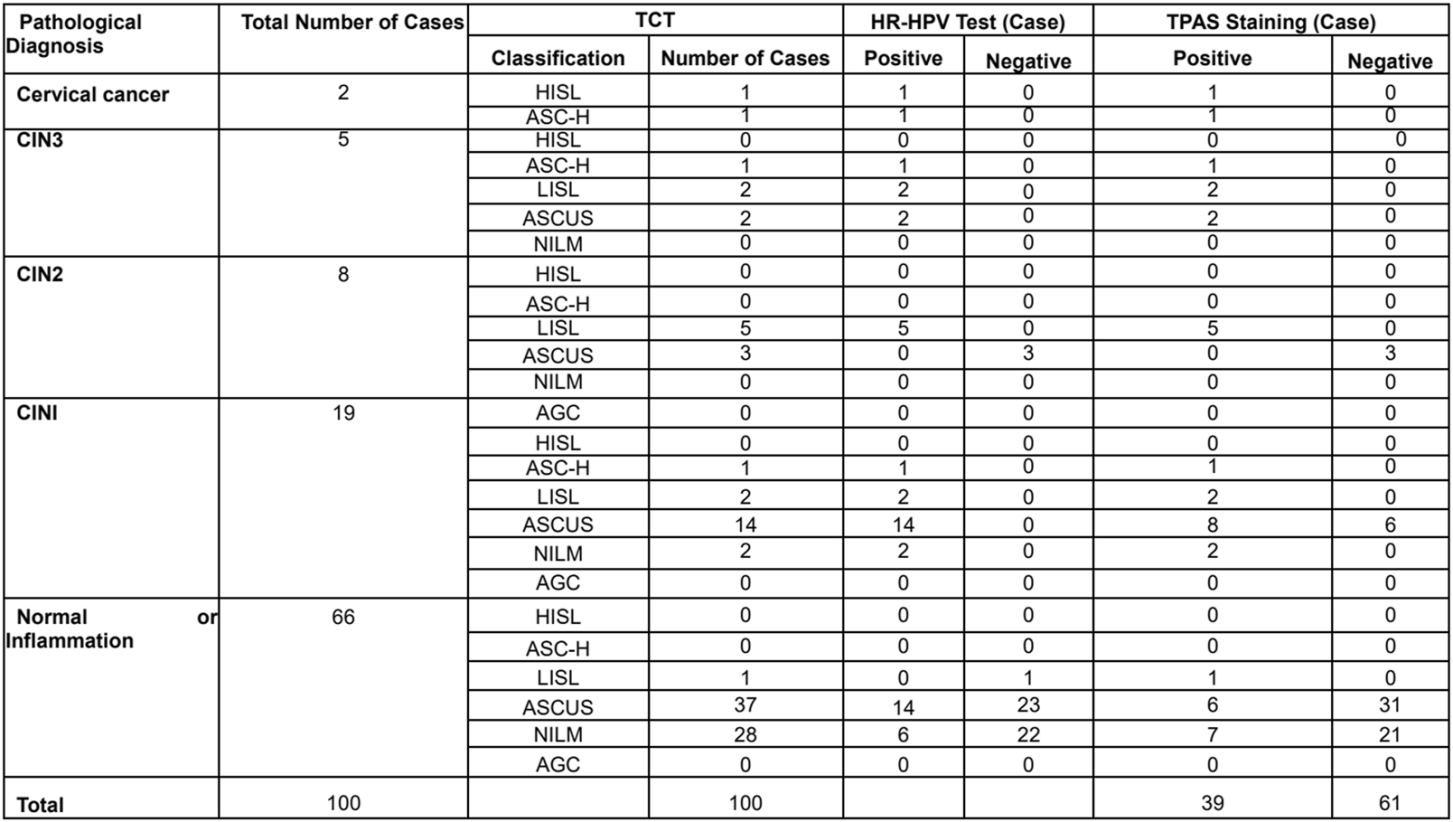
Results of TCT, HR-HPV Test, and TPAS Staining of Women with Different Grades of Precancerous Lesion

*Abbreviations*: *TCT* cervical liquid-based cytology, *HR-HPV* the HR-HPV test, *HSIL* high-grade squamous intraepithelial lesions, *LSIL* low-grade squamous intraepithelial lesions, *AGC* atypical glandular cells, *ASC-H* atypical squamous cells when high-grade squamous intraepithelial lesions could not be ruled out, *ASCUS* atypical squamous cells of undetermined significance, *NILM* no intraepithelial lesion or malignancy

The resultant 100 women were used to calculate clinical performance of TPAS and cytology. For the screening performance in the 100 women, TPAS had general agreement with cytology thresholded at ASC-H (κ-value = 0.33), but had poor agreement with cytology thresholded at ASCUS (κ value = −0.548). The detection rate of TPAS and cytology for different histopathological diagnosis is summarized in Table 2. For normal/inflammatory cases, TPAS showed a much higher DR (80.30%) compared to ≥ ASCUS (42.40%), though this may indicate higher false - positive rates in benign samples.For high - grade lesions (CIN2, CIN3, Cancer), both TPAS and ≥ ASCUS achieved high DR (≥ 75%), with TPAS matching or approaching the sensitivity of ≥ ASCUS cytology.

**Table 2.**
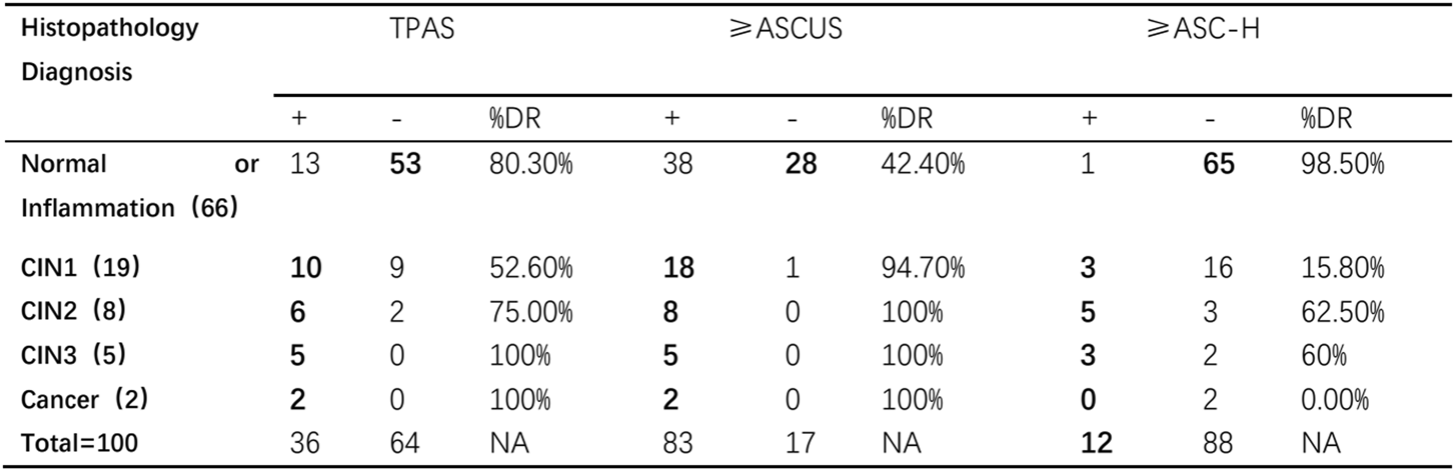
TPAS and cytology results and corresponding detection rate by severity of histopathology diagnosis

*TPAS* TPAS staining, *ASC-US* atypical squamous cells of undetermined significance, *ASC-H* atypical squamous cells, unable to exclude high grade intraepithelial lesion, *CIN* cervical intra-epithelial neoplasias, *DR* detection rate

The ≥ ASC - H threshold had very low DR for low - grade lesions (e.g., 15.80% for CIN1) but high specificity for normal/inflammatory cases (98.50% negative).

Eventually, 100 patients were included in the cervical cancer screen to compare the efficacy of different screening schemes. The sensitivities of TPAS staining, TCT, and the HR-HPV test in the diagnosis of CIN2 and CIN3 were 86.7%, 66.7%, and 80.0%, respectively, and the corresponding specificities were 72.9%, 35.3%, and 54.1%. The sensitivities of HPV + TPAS, and HR-HPV + TCT were 80% and 80%, respectively, with corresponding specificities of 70.6% and 54.1% (Table 3).

**Table 3.**
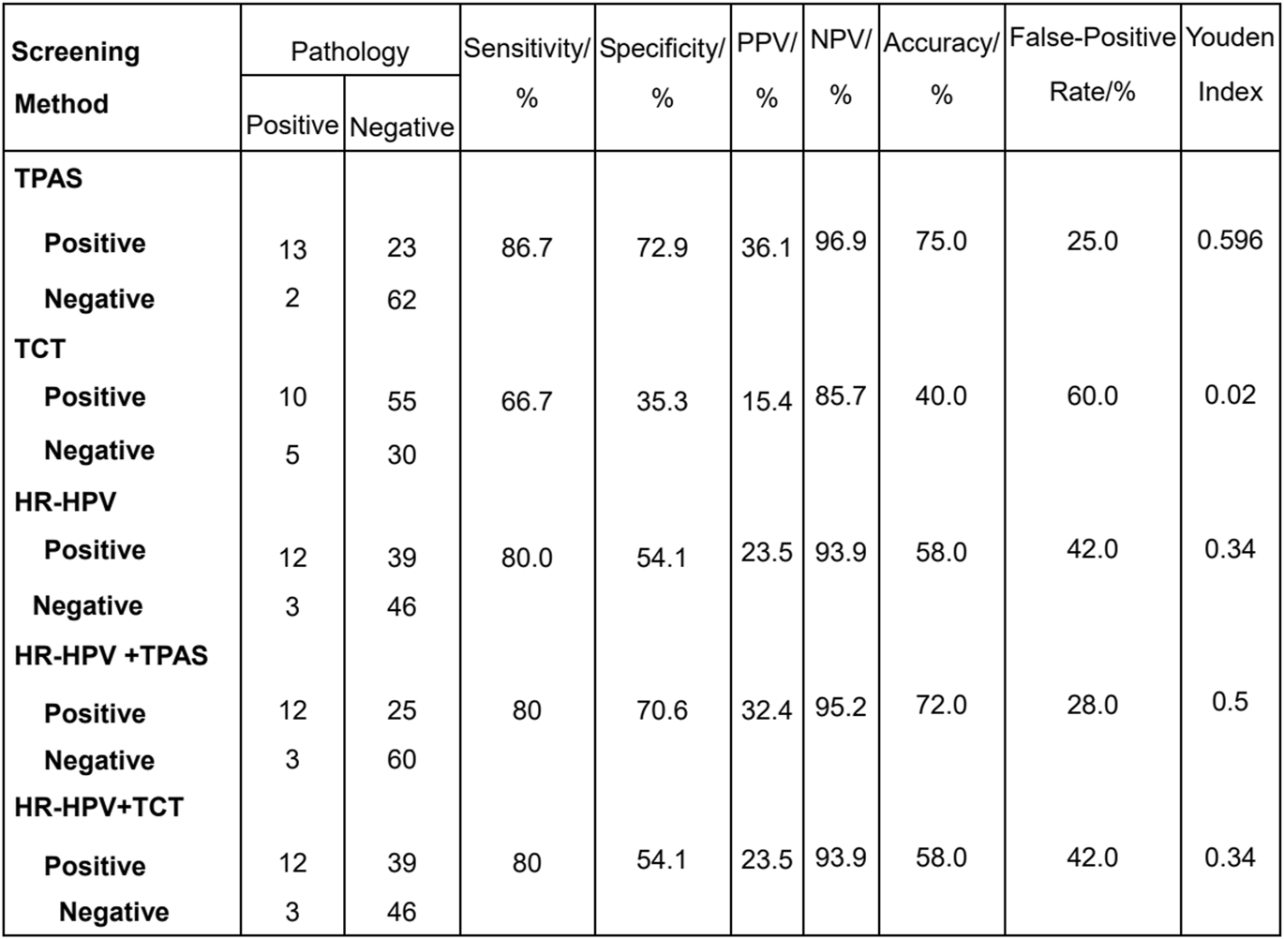
Evaluation of the Clinical Performances of TPAS Staining, TCT, or HR-HPV Test Alone for Cervical Precancerous Lesion Screening and Their Paired Combinations for Screening for Cervical Precancerous Lesions with CIN2+ (HISL) as the Endpoint

### Characterization of TPAS binding and fluorescence intensity in cervical - associated cell lines

**Fig. 1:**
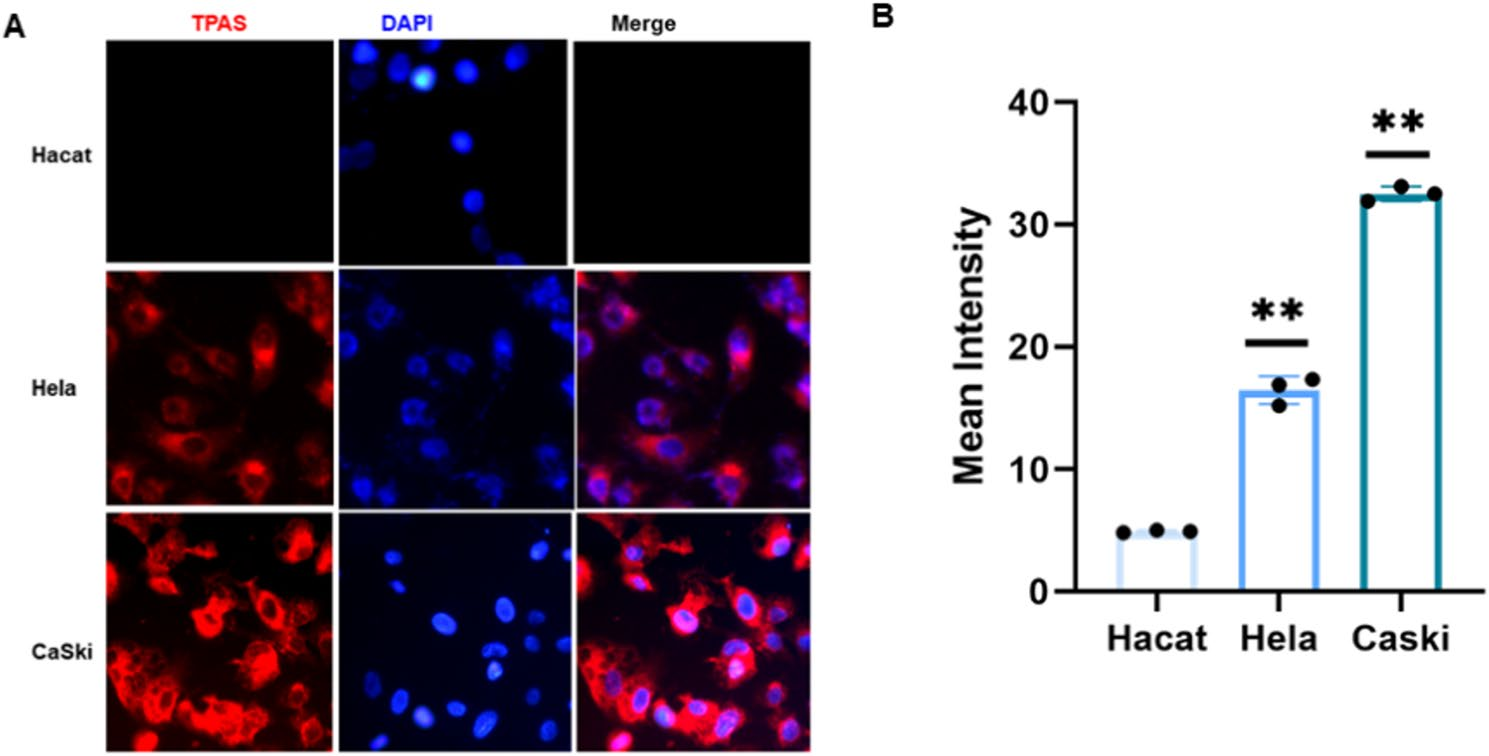
Immunofluorescence of TPAS in normal and cancer cervical cell lines. **A**. Immunofluorescence staining of TPAS (red) in HaCat, HeLa, and CaSki cells. Nuclei were counterstained with DAPI (blue). “Merge” shows the combined fluorescence channels. Scale bar **(**50 μm**)** applies to all panels. **B**. Quantitative analysis of TPAS mean fluorescence intensity in HaCat, HeLa, and CaSki cells. Data represent mean ± SD (*n* ≥ 3 independent experiments). *P* < 0.01 vs. HaCat (one - way ANOVA with post - hoc test)

Fluorescence microscopy was employed to examine the expression levels of TPAS in three distinct cell lines: Hacat, Hela, and CaSki. As depicted in Fig. 1A, DAPI was utilized to stain the nuclei, appearing as blue fluorescence, while TPAS was labeled to exhibit red fluorescence, with merged images showing the colocalization of both.In the Hacat cell line, the red fluorescence intensity of TPAS was extremely low, nearly undetectable, indicating minimal expression of TPAS. In contrast, both the Hela and CaSki cell lines, which are associated with cervical cancer, displayed red fluorescence signals for TPAS. Notably, the CaSki cell line, representing metastatic cervical cancer cells, showed a significantly stronger red fluorescence intensity of TPAS compared to the Hela cell line. The fluorescence in CaSki cells was more intense and widespread, suggesting a higher expression level of TPAS in metastatic cervical cancer cells in Fig. 1B. This result indicates that TPAS may play a more prominent role in metastatic cervical cancer, potentially serving as a biomarker or a target for further investigation in the context of cervical cancer metastasis.

**Fig. 2:**
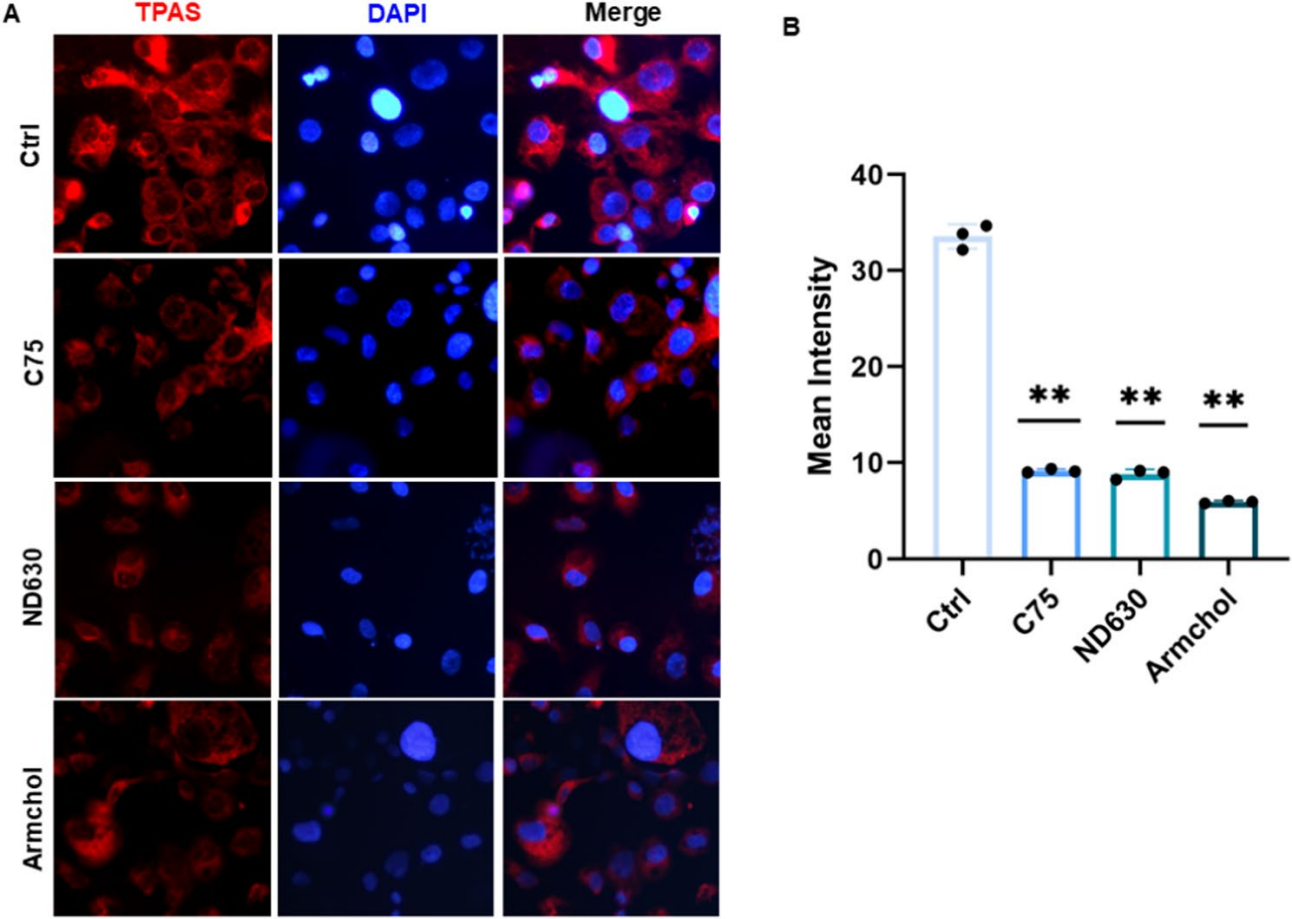
Immunofluorescence of TPAS in cervical cancer cells treated with fatty acid-related inhibitors. **A**. Immunofluorescence detection of TPAS (red fluorescence) in cells under different treatments (Ctrl, C75, ND630, Armchol). DAPI (blue) stains cell nuclei, and “Merge” shows the overlay of TPAS and DAPI signals. Scale bar **(**50 μm**)** applies to all panels. **B**. Quantitative analysis of TPAS mean fluorescence intensity for each treatment group. Data are presented as mean ± SD (*n* ≥ 3 independent experiments). **P < 0.01** vs. Ctrl group (one - way ANOVA with post - hoc test)

To investigate the role of fatty acid metabolism in TPAS labeling in CaSki cells, we treated the cells with fatty acid - related inhibitors. As shown in Fig. 2A, fluorescence microscopy images were taken, where TPAS was labeled with red fluorescence and DAPI was used to stain the nuclei with blue fluorescence, and merged images presented the co - localization of both.The control group (Ctrl) showed a relatively high intensity of TPAS red fluorescence. After treatment with the fatty acid - related inhibitors C75, ND630, and Armchol, the red fluorescence intensity of TPAS in CaSki cells was significantly reduced compared to the control group. Figure 2B further quantified this observation. The bar chart depicted the mean intensity of TPAS fluorescence, revealing that the mean intensity values in the C75, ND630, and Armchol treatment groups were all notably lower than that in the Ctrl group (*P* < 0.01).These results suggest that TPAS labeling in tumor cells is influenced by fatty acid metabolism. Inhibition of fatty acid metabolism pathways by these inhibitors leads to a decrease in TPAS fluorescence intensity, indicating that fatty acid metabolism may play a crucial role in the process of TPAS labeling in CaSki cells.

### TPAS fluorescence patterns and quantified intensity in cervical lesion progression

**Fig. 3:**
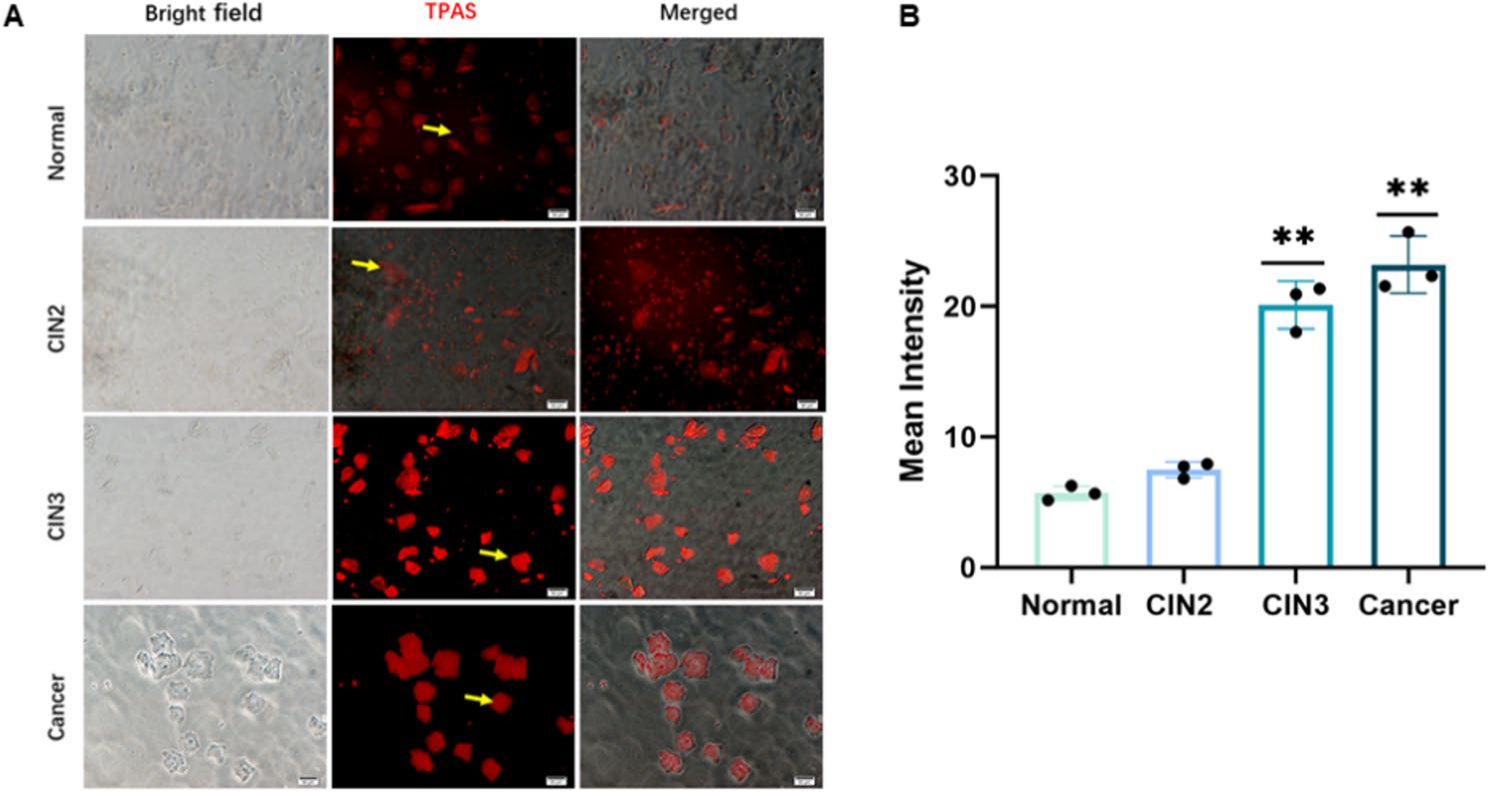
Immunofluorescence of TPAS in normal, CIN2, CIN3, and cancer cervical epithelium. **A** Immunofluorescence analysis of TPAS in normal, CIN2, CIN3, and cancer cervical epithelium. “Bright field” shows cellular morphology under visible light; TPAS staining (red) highlights target protein distribution, with yellow arrows indicating positive signals; “Merged” combines bright - field and TPAS fluorescence images. Scale bar (50 μm) applies to all panels. **B**. Quantitative assessment of TPAS mean fluorescence intensity across groups. Data represent mean ± SD (n ≥ 3 samples per group). P < 0.01 (one - way ANOVA with post - hoc tests for inter - group comparisons)

Fluorescence microscopy was used to analyze the TPAS labeling in cervical exfoliated epithelial cells across different groups: Normal, CIN2, CIN3, and Cancer. In Fig. 3A, bright - field images provided a view of cell morphology, TPAS was visualized with red fluorescence, and merged images combined both aspects. Yellow arrows in the TPAS channel pointed to representative areas of fluorescence.Quantitatively, Fig. 3B shows the mean intensity of TPAS fluorescence. The mean intensity in the Normal group was relatively low. As the CIN grade increased from CIN2 to CIN3, and further in the Cancer group, the mean intensity of TPAS fluorescence significantly increased. Statistical analysis indicated that the differences between the CIN3 and Cancer groups compared to the Normal group were highly significant (*P* < 0.01).

These results suggest that TPAS fluorescence intensity is closely associated with the CIN grading. Higher CIN grades, including CIN3 and cancerous states, exhibit notably stronger TPAS fluorescence, indicating that TPAS may serve as a potential biomarker for evaluating the severity of cervical intraepithelial neoplasia and the progression to cervical cancer.

## Discussion

Numerous studies have emphasized that cervical cancer stands as the only gynecological malignancy with established potential for early diagnosis and curability [12–14]. In the context of cervical cancer prevention and treatment, the early identification of pre - cancerous lesions emerges as a crucial and fundamental aspect. Detecting these precancerous changes at an early stage is pivotal for effectively intervening in the development of cervical cancer, offering the possibility to halt the disease progression and improve patient prognosis.

The progression from persistent high-risk HPV infection to cervical precancer or cancer, a natural history spanning 5 to 15 years, underscores the critical role of effective screening in reducing morbidity and mortality [15] and highlights the window of opportunity for such interventions [11]. The World Health Organization’s 2020 Global Strategy for Cervical Cancer Elimination aims to achieve 70% coverage of high-quality screening among women aged 35–45 by 2030 [16]. However, recent national data (2019) show that China’s screening coverage for women aged 30–49 remains at 36.8%, significantly below the global target [17]. The 2019 guidelines from the American Society for Colposcopy and Cervical Pathology (ASCCP) endorse HPV genotyping as the primary screening modality [18]. While the 2019 ASCCP guidelines endorse HPV genotyping as the primary screening modality [14], China’s clinical practice relies on HPV testing, cytology, or co-testing [19]. This gap is further accentuated by regional disparities: Available evidence suggests that screening rates in rural areas are lower than those in urban centers, though specific data on resource allocation disparities are needed to quantify this divide [15].

Previous studies have reported that the sensitivity of TCT for detecting cervical intraepithelial neoplasia (CIN) 2 + ranges from 55% to 80% [20]. Notably, our study documented a sensitivity of 66.7%—a value that aligns with the lower spectrum of published data, potentially reflecting regional variations in sample demographics or diagnostic criteria. This discrepancy is rooted in interobserver variability among pathologists: highlights that inconsistent morphological interpretation leads to inherent rates of missed diagnosis (e.g., false negatives for high-grade lesions) and overdiagnosis (e.g., false positives for benign atypia).

Beyond diagnostic inconsistency, TCT imposes substantial operational burdens [21, 22], underscores that automated liquid-based cytology systems entail equipment costs exceeding $50,000 per facility and require 2–3 certified cytotechnologists per 10,000 tests, creating a bottleneck in resource-constrained settings. These infrastructure demands translate to prolonged turnaround times (median 7–14 days in rural hospitals), which contribute to high patient dropout rates. Cumulatively, these factors—technical variability, economic cost, and logistical inefficiency—hinder the scalability of TCT for population-level screening.

HPV testing is characterized by high sensitivity, high negative predictive value, and good repeatability. However, most HPV infections are transient and naturally cleared within 1–2 years. Therefore, HPV testing may lead to overdiagnosis, increasing the false-positive rate and colposcopy referral rate, which can cause patient anxiety. Moreover, HPV testing can only detect HPV infection but cannot determine the degree of cervical lesions [23, 24].

Our cell line studies provided insights into the biological function of TPAS. In the Hacat cell line, which is a normal keratinocyte cell line, the expression of TPAS was extremely low. In contrast, in cervical cancer - related cell lines (Hela and CaSki), TPAS was expressed, and it was notably higher in metastatic CaSki cells. This suggests that TPAS may be specifically associated with the carcinogenesis and metastasis of cervical cancer. The finding that fatty acid - related inhibitors could reduce the TPAS fluorescence intensity in CaSki cells implies that fatty acid metabolism plays a role in TPAS labeling. This may open up new research directions in understanding the metabolic - associated mechanisms of TPAS in cervical cancer cells.

In terms of clinical screening, TPAS demonstrated a moderate agreement with cytology when the threshold was set at ASC-H, while showing poor agreement with cytology at the ASCUS threshold. The sensitivity of TPAS staining for diagnosing CIN2 and CIN3 was 86.7%, which was higher than that of TCT (66.7%) and comparable to the HR-HPV test (80.0%). The specificity of TPAS staining (72.9%) was also more favorable compared to TCT (35.3%). These findings suggest that TPAS has potential as a screening tool for cervical intraepithelial neoplasia. The combination screening schemes, such as HPV + TPAS and HR-HPV + TCT, did not show a significant improvement in sensitivity compared to individual tests, but maintaining certain levels of specificity. This indicates that while combination strategies can be considered, further optimization may be needed to enhance their overall performance.

Regarding the analysis of cervical exfoliated epithelial cells, TPAS fluorescence intensity was closely related to the CIN grading. As the CIN grade increased from CIN2 to CIN3 and to cancer, the TPAS fluorescence intensity significantly increased. This indicates that TPAS has the potential to be a biomarker for evaluating the severity of cervical intraepithelial neoplasia and the progression to cervical cancer. It could potentially assist in the early detection and monitoring of cervical lesions.

However, this study has some limitations. The sample size was relatively small, which may limit the generalizability of the results. Future studies should aim to include a larger number of patients to confirm these findings. Additionally, the underlying molecular mechanisms of TPAS in cervical cancer, especially its relationship with fatty acid metabolism, need further exploration. Limitations of the TPAS test include that it only indicates color changes, which cannot assess the severity of cervical lesions and is relatively subjective. Furthermore, research on the use of TPAS testing for clinical screening is currently limited and requires further investigation to validate its effectiveness.This study aimed to evaluate the clinical performance of TPAS in cervical cancer screening and explore its biological characteristics in different cell lines and cervical exfoliated epithelial cells.

## Conclusion

In conclusion, TPAS emerges as a promising biomarker for cervical cancer screening, with potential biological relevance in elucidating cervical cancer pathogenesis. While our findings highlight its strengths in sensitivity, specificity, and lesion severity stratification, further research is indispensable. Large - scale trials, mechanistic investigations, and optimization of clinical workflows are needed to fully realize TPAS’s potential and integrate it effectively into clinical practice. By addressing these avenues, we can move closer to enhancing cervical cancer screening precision and ultimately improving patient outcomes.

## Data Availability

No datasets were generated or analysed during the current study.

## Abbreviations

AGC: Atypical glandular cells
ASC-H: Atypical squamous cells, unable to exclude high grade intraepithelial lesion
ASC-US: Atypical squamous cells of undetermined significance
CIN: Cervical intra-epithelial neoplasias
DR: Detection rate
HPV: Human papillomavirus
HSIL: High-grade squamous intraepithelial lesions
LBC: Liquid-based cytology
LSIL: Low-grade squamous intraepithelial lesions
NILM: No intraepithelial lesion or malignancy
TCT: Thin-layer cytological testing
TPAS: Viscosity-responsive plasma membrane probe
